# Surveillance of HCC Patients after Liver RFA: Role of MRI with Hepatospecific Contrast versus Three-Phase CT Scan—Experience of High Volume Oncologic Institute

**DOI:** 10.1155/2013/469097

**Published:** 2013-11-12

**Authors:** Vincenza Granata, Mario Petrillo, Roberta Fusco, Sergio Venanzio Setola, Elisabetta de Lutio di Castelguidone, Orlando Catalano, Mauro Piccirillo, Vittorio Albino, Francesco Izzo, Antonella Petrillo

**Affiliations:** ^1^Division of Radiology, Department of Diagnostic Imaging, Radiant and Metabolic Therapy, “Istituto Nazionale Tumori Fondazione Giovanni Pascale-IRCCS”, Via Mariano Semmola, 80131 Naples, Italy; ^2^Division of Hepato-Biliary Surgery, Department of Abdominal Oncology, “Istituto Nazionale Tumori Fondazione Giovanni Pascale-IRCCS”, Via Mariano Semmola, 80131 Naples, Italy

## Abstract

*Purpose*. To compare the diagnostic accuracy of hepatospecific contrast-enhanced MRI versus triple-phase CT scan after radiofrequency ablation (RFA) in hepatocellular carcinoma (HCC) patients. *Methods*. Thirty-four consecutive HCC patients (42 hepatic nodules) were treated with percutaneous RFA and underwent MR and CT scans. All patients were enrolled in a research protocol that included CT with iodized contrast medium injection and MR with hepatospecific contrast medium injection. All patients were restaged within four weeks and at 3 months from ablation. The images were reviewed by four different radiologists to evaluate tumor necrosis, residual or recurrence disease, and evidence of new foci. *Results*. Thirty-two nodules were necrotic after treatment; 10 showed residual disease. Six new HCCs were identified. At first month followup CT has identified 34 necrotic lesions and 8 residual diseases; no new foci were recognized. At MRI instead, 32 complete necrotic lesions were identified, 10 lesions showed residual disease, and 2 new HCCs were found. At three months, CT demonstrated 33 completely necrotic lesions, 9 residual diseases, and 2 new HCCs. MR showed 31 complete necrotic lesions, 11 cases of residual disease, and 6 new HCCs. *Conclusions*. Hepatospecific contrast-enhanced MRI is more effective than multiphase CT in assessment of HCC treated with RFA.

## 1. Introduction

Hepatocellular carcinoma (HCC) is one of the most common causes of cancer death worldwide [1]. Although surgical resection offers a better curative option than nonsurgical treatments, it is not an option for the majority of patients because of the presence of poor hepatic function and the typically advanced nature of the disease at presentation [2].

Effective nonsurgical treatment of HCC includes radiofrequency ablation (RFA). Several randomized controlled trials have demonstrated that RFA is able to achieve higher rates of complete tumor ablation using fewer sessions, with tumor necrosis rates of 90–95% in solitary HCC under 4 cm [3–7]. Assessing the effectiveness of RFA is critical in determining the success of treatment and in guiding future therapy. However, current imaging modalities and imaging response criteria are limited in their ability to provide clinically satisfactory information about the extent of tumor necrosis, which is essential in determining patient prognosis [8].

In 2000, have been established common parameters to define cancer response to therapy by means of the introduction of the Response Evaluation Criteria in Solid Tumors (RECIST). RECIST 1.1, published in January 2009, was an update to the original criteria [3]. Both criteria were based on decrease in tumor size as evidence of successful therapy [9] resulting inadequate when new antcancer therapies are considered, being the response, quantified in terms of tumor necrosis, and not often correctly evaluated through the “one-dimensional assessment” of treated tumors. Such difficulties emerged with the introduction of regional therapies, leading to the development of new imaging evaluation criteria focused on tumor vascularization assessment, cell necrosis, mobility of water molecules, and concentration of particular metabolites in the context of tumor, pointing to a multiparametric evaluation that could add more accurate data than “one-dimensional” assessment. European Association for the Study of the Liver (EASL), in accordance with the guidelines of the American Association for the Study Liver Diseases (AASLD), recommended the assessment of lesion contrast enhancement through an MDCT examination, as “standard approach” to evaluate HCC response to therapy after local regional therapies.

The decrease in viable cell mass is not necessarily reflected by changes in tumor size, and tumors may not decrease in size after RFA despite the fact that they are nonviable [10, 11]. Tumors may apparently increase in size, and this is caused by inclusion around the treated lesion of a safety margin to improve clinical ablation success [11]. CT is the most widely used modality to confirm the technical success of RFA [12] even if MRI due to its intrinsic imaging capabilities and the absence of radiation exposure has been well shown as an alternative candidate to MDCT examination. The availability of biphasic contrast media, such as gadoxetate disodium, with a combined vascular and an elective hepatocytic uptake, is potentially able to offer more chance to standardize HCC followup after treatment, offering at the same time a morphological imaging combined with a purely functional imaging [4]. Gadoxetate disodium is a gadolinium-based liver specific MR contrast agent that differs from most of other gadolinium agents in possessing substantially increased r1 relaxivity in blood, which can be used to either reduce the dose of contrast medium or increase the degree of both vascular and parenchymal contrast enhancement. Furthermore, since about half of the gadoxetate disodium amount is eliminated through the hepatobiliary pathway, liver-specific imaging during the delayed hepatobiliary phase can be performed to improve both lesion detection and lesion characterization. 

Moreover, according to EASL criteria, MRI for its intrinsic contrast resolution is particularly suitable in “identification” and “quantization” of the necrosis induced by ablative therapies. Liver-specific MR contrast agents having different pharmacokinetics, compared to traditional gadolinium chelates, opened new interesting perspectives in terms of diagnostic accuracy particularly when residual active tumor, “benign enhancement,” and perilesion tissue must be differentiated; in the context of a cirrhotic liver new malignant nodules can develop. MRI with liver-specific contrast agent can be used to confirm the technical success of RFA [13].

Aim of this study was to evaluate the diagnostic accuracy of magnetic resonance imaging (MRI) by liver-specific contrast agent, gadoxetic acid (Gd-EOB-DTPA, Primovist), when compared with multidetector computed tomography (MDCT) after radiofrequency ablation (RFA), to assess the evidence of residual tumor tissue.

## 2. Materials and Methods

### 2.1. Patients

This study was approved by our institutional review board, and written informed consent was obtained from all patients. Between March 2009 and May 2010, 34 consecutive patients with 42 pathologically proven HCCs (diameter between 16 and 40 mm) were treated with RFA under sonography guide (Table 1).

All RFA treatments were carried out with the patient under general anesthesia and tracheal intubation, in the operating room, under sonographic guidance. One grounding pad was placed on the posterior surface of each thigh. A 250 W monopolar instrument (Radiotherapeutics, Mountain View, CA, USA), with a 12-hook 14 G electrode (LeVeen needle electrode) generating a 4 cm array diameter, was used. RFA treatment consisted typically of two phases with a one-minute cooling interval. The first RFA application lasted about 7 minutes while the second lasted about 2 minutes. 

### 2.2. Imaging Technique

All patients underwent both a triple-phase MDCT and MR examinations, before RFA, at 1 M and at 3 M after RFA. Contrast-enhanced triple-phase helical MDCT was performed with a 16-detector row scanner (Brilliance 16, Philips Medical Systems, Eindhoven, the Netherlands). MDCT scanning parameters were 120 kVp, 189–200 mAs, 5 mm slice thickness with an increment (overlap) of 2.5 mm, and table speed of 18.75–26.75 mm/rotation (pitch 0.828–1.07). Scans were performed in craniocaudal direction. Scans were carried out including a region encompassing liver from diaphragm dome to iliac crests as follows: hepatic arterial phase scanning began 30–40 s after injection of 120 mL of a nonionic iodinated contrast media (CM, iomeprol, Iomeron 400, Bracco, Italy) at a rate of 4 mL/s with a bolus-triggered technique (120 kVp; 40–60 mA; monitoring frequency from 12 seconds after the contrast injection; trigger threshold, 100 HUs in descending aorta; delay from trigger to initiation of scan, 18 seconds); portal and equilibrium phases were obtained scanning the same region respectively 70 seconds and 180 seconds after CM injection. CM was administered through antecubital vein with an automated injector system (Empower CTA, E-Z-EM Inc., New York, NY).

MRI examination was performed with a 1.5-T MR system (Magnetom Symphony, upgraded to Total Imaging Matrix Package, Siemens, Erlangen, Germany) with an eighteen-channel body surface phased-array coil. The liver was imaged in the axial plane in all patients both before and after administration of gadoxetic acid (Primovist, Bayer Schering Pharma, Germany) at a dose of 0.1 mL/kg (0.25 mmol/mL). The contrast agent was administered IV at a rate of 2 mL/s followed by a 20 mL saline flush through the antecubital vein with a power injector (Spectris Solaris EP MR; MEDRAD, Inc., Indianola, PA).

The MRI protocol included a respiration triggered T1-weighted turbo field-echo in-phase and out-of-phase acquisition (TR/TE 160/2.35–4.87 ms, flip angle 70°, slice thickness 5 mm, gap 20%, base resolution 256 mm, phase resolution 90%, and parallel imaging using Generalized autocalibrating partially parallel acquisition (GRAPPA) with acceleration factor 2, acquisition time 33 s) and a breath-hold multishot T2-weighted acquisition with and without fat suppression (TR/TE 1500/90 ms, flip angle 170°, slice thickness 5 mm, gap 0 mm, base resolution 320 mm, phase resolution 78%, and GRAPPA with acceleration factor 2, acquisition time 45 s). For gadoxetic acid-enhanced MRI, unenhanced, arterial phase (20–35 s), portal phase (70 s), equilibrium phase (3 min.), and delayed hepatobiliary phase (20 min.) images were obtained with a T1-weighted 3D turbo-field-echo sequence (T1 high-resolution isotropic volume examination, Vibe, Siemens Healthcare) (TR/TE 4.80/1.76 ms, flip angle 12°, slice thickness 3 mm, gap 20%, base resolution 320 mm, phase resolution 70%, and GRAPPA with acceleration factor 2, acquisition time 18 s).

### 2.3. Images Review

Four blinded observers with at least 7 years' experience in interpretation of MR and CT images of the liver independently and randomly reviewed the CT and MR images acquired at one and at three months after RFA. The four reviewers had all been involved in the original studies. The interval between reviews of the CT and MR images was at least 15 days. The observers recorded in consensus the presence and segmental location of the treated lesions, using a 4-point confidence scale (score) based on published studies on HCC [14]: 1, no residual HCC; 2, probably no residual HCC; 3, probable residual HCC; 4, definite residual HCC. They also evaluated the presence of new HCC using the same 4-point confidence scale both for arterial phase, portal phase, and hepatobiliary phase. In clinical practice at our institution, nodules that become enhanced in the arterial phase and show a washout pattern in the portal or equilibrium phase with or without capsular enhancement at triple-phase MDCT are considered HCC. So treated lesions that show an area that becomes enhanced in the arterial phase and have a washout pattern in the portal or equilibrium phase are considered still active lesions. Completely ablated lesions appear as a hypoattenuating area with no foci of contrast enhancement either within the lesion or at its periphery. Moreover, for RF ablation to be complete, the entire tumor as well as a peripheral safety margin of 0.5–1 cm of normal hepatic tissue must be ablated.

The criteria for residual HCC and new foci of HCC on gadoxetic acid-enhanced MR images were similar to the criteria for the triple-phase CT pattern. In addition, a hypointense nodule seen on gadoxetic acid-enhanced hepatobiliary phase MR images was considered HCC on the basis of previous findings [15]. A hypervascular nodule seen on gadoxetic acid-enhanced arterial phase MR images with a washout pattern was considered HCC even though the nodule appeared isointense or hyperintense relative to the surrounding liver parenchyma on hepatobiliary phase images [15]. On unenhanced T1- and T2-weighted MR imaging, the signal intensity within the ablated lesion was reported too.

The standard reference for diagnosis of complete necrosis was considered the subsequent CT and MR imaging, showing persistent absence of contrast enhancement. All cases with residual tumor tissue or with new nodules underwent biopsy. 

### 2.4. Statistical Analysis

Sensitivity, specificity, negative predictive value and positive predictive value were reported for both imaging techniques. Fischer tests were used in order to evaluate statistical significance of table 2 × 2 for CT and MRI [16]. Statistical analyses for the differences of calculated sensitivity and specificity values of TC and RM were performed using McNemar test [17]. Statistical analyses for the differences of calculated positive and negative predictive values for each observer and technique were performed as previous report [17]. An analysis of all false positive and falsenegative observations was also undertaken. 

The alternative free-response receiver operating characteristic (ROC) analysis of all lesions was performed tumor by tumor [18]. The area under the ROC curve, computed using trapezoid rule integration, was used to assess the diagnostic accuracy of each technique. A *P* value <0.05 was considered to indicate a statistically significant difference. Statistical analysis was performed using statistic toolbox of Matlab R2007a (Matworks, Natick, MA).

## 3. Results

**Figure 1:**
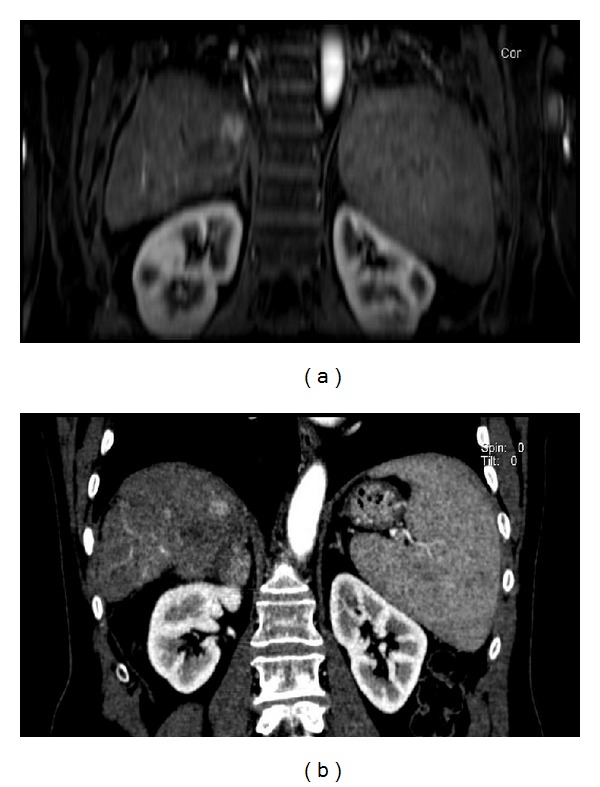
77-year-old man treated with RFA for HCC. (a) Arterial-phase, coronal Vibe T1-W FS at 1-month followup. Residual tumor tissue in the cephalad portion of the treated area. (b) Coronal reformatted, artery-phase CT at 1-month followup. Residual tumor tissue in the cephalad portion of the treated area.

**Figure 2:**
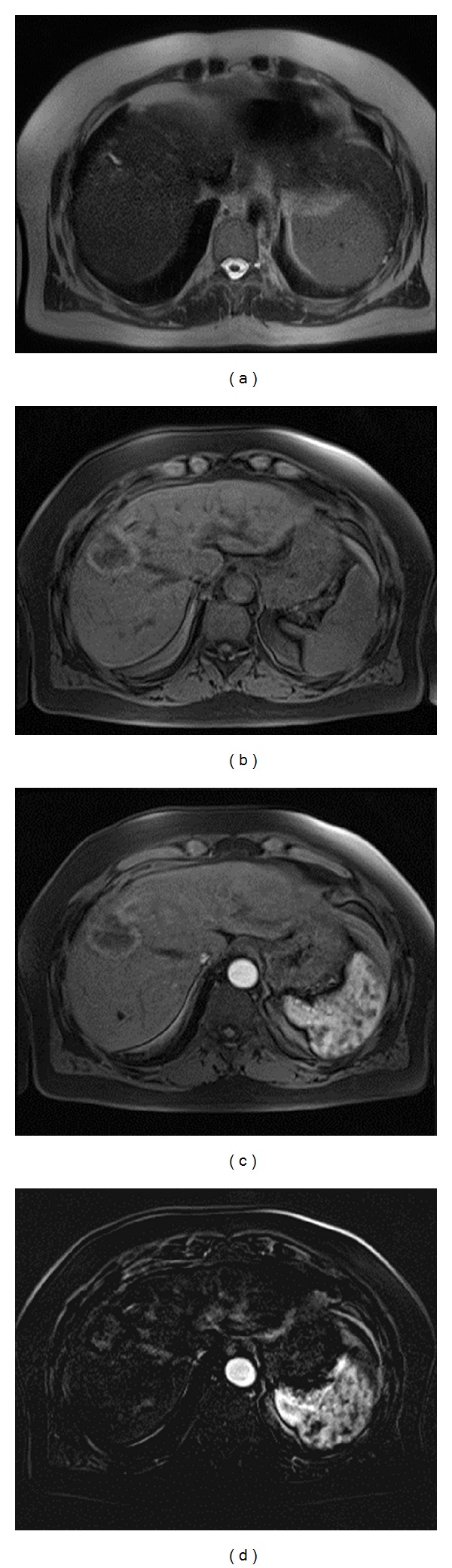
69-year-old woman treated with RFA for HCC. (a) Axial Haste T2-W, at 1-month followup; treated necrotic nodule, heterogeneously isointense to hypointense. A dilatation of an intrahepatic biliary branch, as a complication of RFA, is recognizable. (b) Axial Vibe T1-w FS, at 1 month followup: treated necrotic nodule, heterogeneously isointense to hyperintense, with peripheral hypointense rim. (c) At 1-month followup arterial-phase scan MR image showing no contrast enhancement. (d) At 1-month followup postprocessing subtract image (arterial phase and no contrast phase) showing no contrast enhancement.

At one month, CT detected 34 necrotic lesions, including 30 true necrotic lesions and 4 false negatives. Additionally, there were 8 residual tumors detected by CT, including 6 true residual tumors (Figure 1(a)) and 2 false positives. CT failed to recognize 2 new HCCs. At 1-month followup, MR detected 32 necrotic lesions, with 31 true necrotic lesions (Figure 2) and 1 false negative. Additionally, there were 10 residual tumor diagnoses, with 9 true residual tumors (Figure 1(b)) and one false positive. MR detected 2 new HCCs. MRI had a higher sensitivity and specificity (McNemar test) than CT, at one month, and the differences between the two techniques were statistically significant (*P* < 0.05) (Table 2). Regarding the positive and negative predictive values, significant differences were seen between the two techniques with *P* < 0.05.

**Figure 3:**
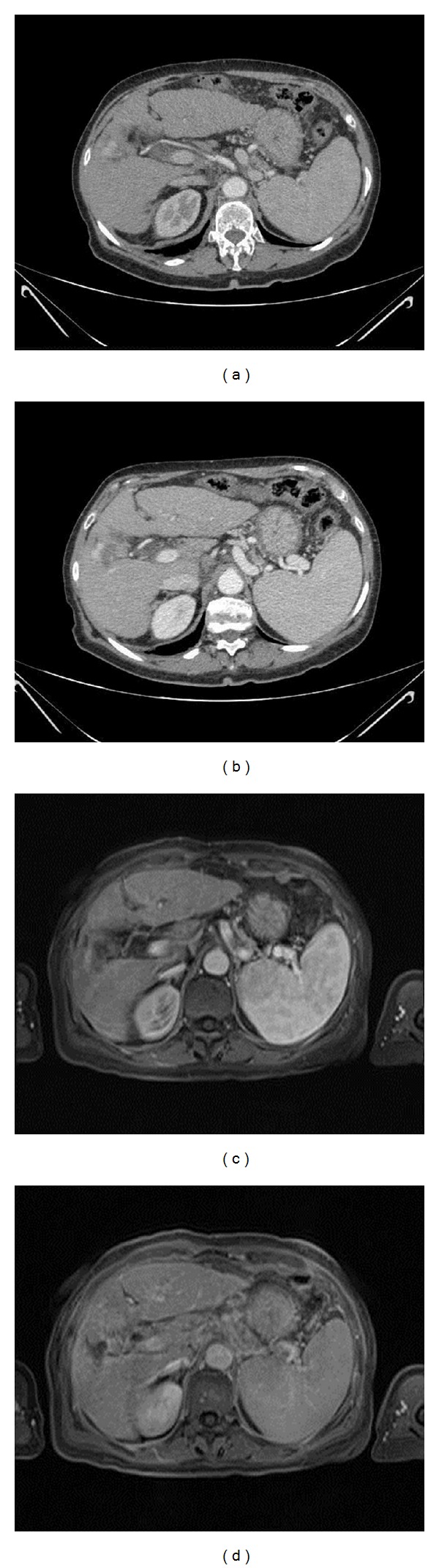
66-year-old woman with an arteriovenous shunt after RFA for HCC. (a) Arterial-phase CT scan at 3-month followup. Enhancing tissue close to the ablated area, incorrectly diagnosed as residual tumor. (b) Portal-phase CT scan at 3-month followup. Enhancing tissue close to the ablated area, incorrectly diagnosed as residual tumor. The lack of washout should have raised the suspicion of a benign finding. (c) Arterial-phase MR image at 3-month followup. Enhancing tissue close to the ablated area, incorrectly diagnosed as residual tumor. (d) Portal-phase MR image at 3-month followup. The lack of wash-out and the same parenchymal enhancement identify the lesion as benign.

**Figure 4:**
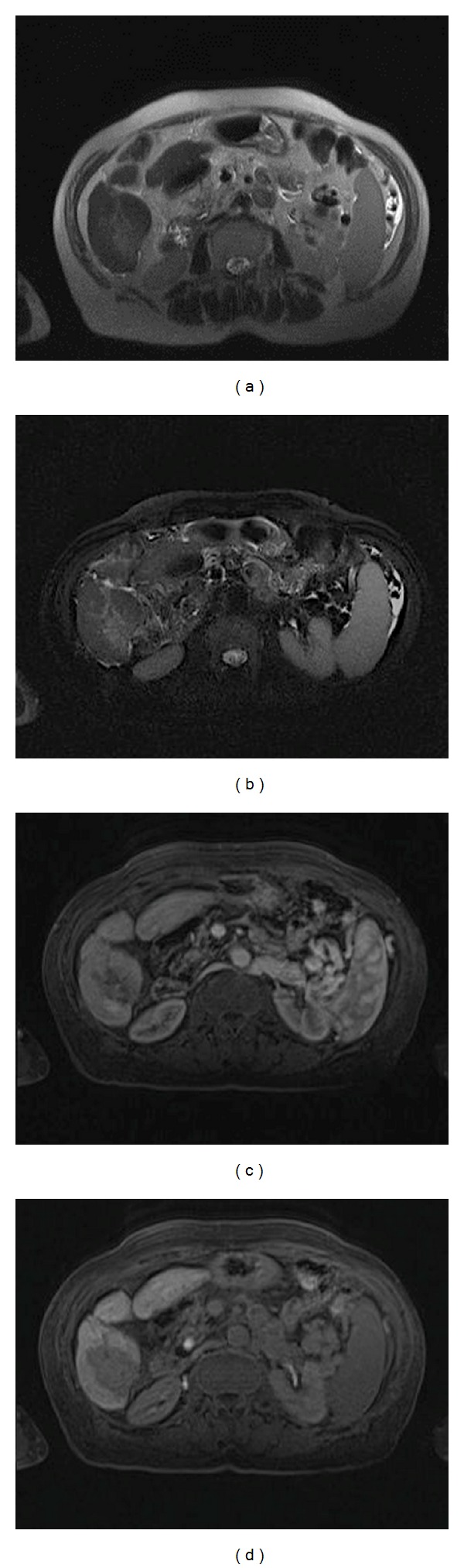
50-year-old man with an ablated HCC at segment 6. At three months, MRI showed that at T2-W (a) and T2-W FS (b) there was no inflammatory reactions while during arterial (c) and hepatospecific phase (d) residual disease was evident.

At 3-month followup, CT detected 33 necrotic lesions with 30 true necrotic lesions and 3 false negatives. There were also 9 residual tumors, with 7 being true residual tumors and 2 being false positive diagnoses (Figure 3). CT recognized 2 new HCC nodules, confirmed at biopsy. At 3-month followup, MR detected 31 necrotic lesions, all being true necrotic lesions with no false negative diagnosis. There were 11 residual tumors with 10 being true residual tumors (Figure 4) and one being a false positive. MRI detected 6 new HCCs, all confirmed at biopsy. The size of these new nodules was 6–12 mm (mean, 10 mm). MRI showed a higher sensitivity and specificity (McNemar test) than CT at three months, and the difference between the two techniques was statistically significant (*P* < 0.05) (Table 3). Regarding the positive and negative predictive values, significant differences were seen between the two techniques with *P* < 0.05.

In Table 4, the CT score was reported to identify the false positive at first and three months at arterial phase and portal phase. At arterial phase CT characterizes both at first and three months all lesions as residual diseases while at portal phase only at three months seen the two lesions as probably residual diseases.

In Table 5, the MR score were reported to identify the false positive at first and three months at arterial phase, portal phase, and hepatobiliary phase. At arterial phase MR characterizes both at first and three months all lesions as residual diseases, at portal phase only one lesion as probably residuals at three months, and at hepatobiliary phase one lesion as probably residual at one month and as probably necrotic area at three months. 

The gadoxetic acid-enhanced hepatobiliary phase MR images allowed identifying false positive better than arterial phase.

The area under curves calculated by ROC analysis were, respectively, for the CT followup at one month 0.98, for the CT followup at 3 months 0.99, for the MR followup at one month 0.99, and for the MR followup at 3 months 1. 

MR showed a major accuracy in the identification of necrotic or residual lesions after RFA having a major area under curve.

In Table 6, the CT and MR scores were reported to identify new HCC at first months at arterial phase, portal phase, and hepatobiliary phase. CT did not see any lesions both at arterial than portal phase. MR characterized one lesion probably as new HCC at arterial phase and two lesions probably as new HCC at portal phase, while at hepatobiliary phase MR characterized both lesions as HCCs.

In Table 7, the CT and MR scores were reported at three months at arterial phase, portal phase, and hepatobiliary phase. CT seen only two lesions (2/6; 33.3%) both at arterial than portal phase. At arterial phase MR did not see two lesions while it characterized one lesions as probably new HCC and three lesions as new HCCs (3/6; 50%). At portal phase MR characterized three lesions as probably new HCC and three lesions as new HCCs (3/6; 50%). At hepatobiliary phase MR characterized each lesion as new HCC (6/6; 100%).

**Figure 5:**
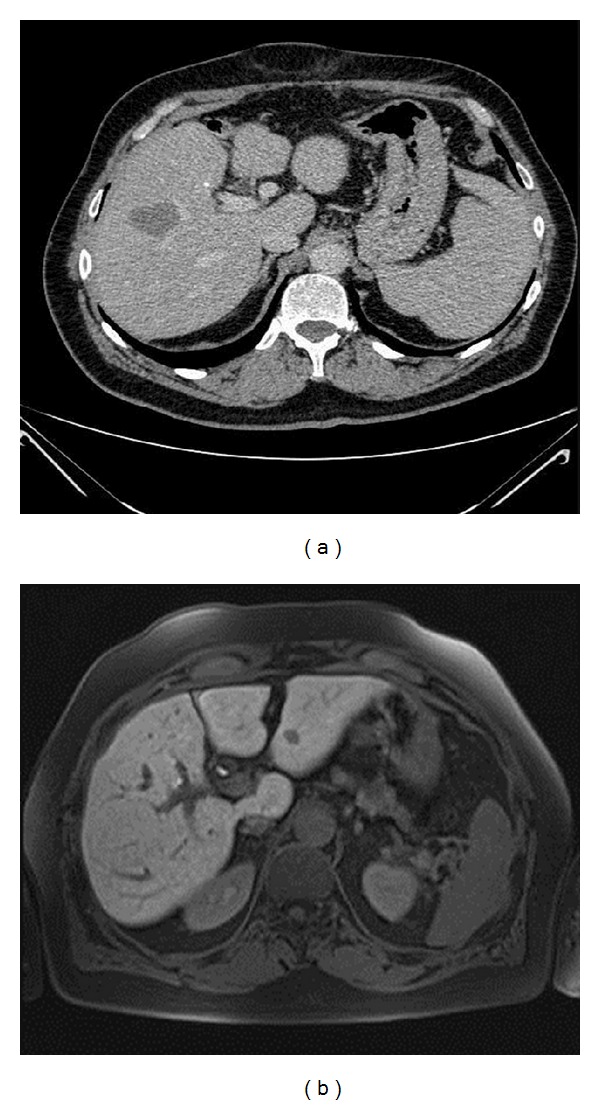
63-year-old man with an ablated HCC at segment 5–8. (a) Portal-phase CT scan at 1-month followup. The necrotic lesion is recognizable. (b) Hepatospecific phase MR image at 1-month followup. A new HCC is evident in the left lobe.

The gadoxetic acid-enhanced hepatobiliary phase MR images allowed identifying new small HCCs better than arterial phase (Figure 5).

## 4. Discussion

Accurate imaging evaluation is important in determining whether a tumor is completely treated or needs additional treatment. Early detection of residual or locally recurrent tumor after RF ablation of HCC is critical and can facilitate successful retreatment at an early stage. Late diagnosis results in peripheral regrowth and makes retreatment difficult owing to limited access [19].

Our study demonstrates that gadoxetate disodium-enhanced MR imaging with combined interpretation of dynamic and delayed hepatobiliary phase images significantly improves the sensitivity in the detection of residual HCC compared to multidetector CT. The diagnostic accuracy improved accordingly due to combined analysis of dynamic and hepatobiliary phase compared to dynamic MR imaging alone and significant improvement for the combined analysis compared with multidetector CT alone.

In our study, at followup CT, all completely ablated lesions appear as a hypoattenuating area with no foci of contrast enhancement either within the lesion or at its periphery; treated areas with focal enhancement during arterial phase and wash-out during portal and equilibrium phase were considered still active tumors. 

Unenhanced T1-W and T2-W MR imaging demonstrates markedly heterogeneous signal intensity within the ablated lesion. This variability in signal intensity, according to previous study, is caused by an uneven evolution of coagulation necrosis and the host response to thermal injury over time [20]. Gadolinium-enhanced dynamic MR imaging is known to be a useful diagnostic method for evaluating therapeutic response after RF ablation of HCC [21]. As at CT, the presence or absence of contrast enhancement in the treated lesion is instructive. A tumor that has been completely treated no longer enhances on gadolinium-enhanced dynamic MR images. When a tumor is not completely treated, residual or recurrent tumor is usually seen as focal and nodular enhancement at the margin of the ablated lesion. During the delayed hepatobiliary phase the residual tumor and the ablated lesion appear as a hypointense area.

In our experience, the residual HCCs were detected and assigned a high confidence score by all readers with the inclusion of delayed hepatobiliary phase MR images.

Along with improvement in sensitivity and diagnostic accuracy, a further advantage of the combined interpretation of dynamic and hepatobiliary phase MR images was the lower number of false positive findings compared with those using dynamic MR or CT image sets, with a consequent increase in positive predictive value. In agreement with previously published findings [22], most of our false positive results were small arteriovenous shunts that were misinterpreted as residual HCC because of their nodular appearance and unequivocal arterial phase enhancement at either multidetector CT or dynamic MR imaging.

With combined interpretation of dynamic and delayed hepatobiliary phase MR images, all arterial-portal venous shunts were correctly assessed by each reader, including two lesions that were prospectively misinterpreted by two readers as residual tumor on dynamic CT images alone.

The advances in imaging technology may also come at the cost of detection of an increased number of hypervascular liver lesions deemed too small to characterize, lesions that might have gone unnoticed in the past. Although most of these small undetermined lesions are nonneoplastic even in patients with pathologically proved HCC [22], in the clinical practice of radiology, any new hepatic lesion discovered at followup imaging in a cirrhotic liver must be assumed to represent an HCC until proved otherwise. On the basis of our findings and similar results [22], we believe that, as an adjunct to dynamic MR imaging, hepatobiliary phase MR images can improve the characterization of most of these undetermined, diminutive lesions.

The four false negative CT examinations were attributed to the reactive hyperemia in tissue surrounding the ablated lesion that hides the residual tumor. The combined interpretation of T2-W, dynamic and hepatobiliary phase MR images has made it possible to identify residual tumor.

Peripheral rim enhancement resulting from reactive hyperemia is usually uniform in thickness and envelops the ablated lesion, whereas residual tumor demonstrates focal and irregular peripheral enhancement. In addition, peripheral rim enhancement representing reactive hyperemia is high or isoattenuating during the portal venous and equilibrium phases. Such reactive hyperemia in tissue surrounding the ablated lesion may make accurate assessment of therapeutic response difficult [21]. 

Moreover, for RF ablation to be complete, the entire tumor as well as a peripheral safety margin of normal hepatic tissue must be ablated. In all ablated lesions, in our study, CT and MRI showed the ablated lesion to be larger than the preablation tumor, while nine residual HCCs had equal size preablation tumors. In only one case residual tumor was placed at the edge of a large treated area.

The results of our study demonstrate also that, compared with multiphasic 16-section multidetector CT, gadoxetate disodium-enhanced MR imaging yields significantly higher diagnostic accuracy and sensitivity for the detection of new HCC in treated patients. Our data corroborate the results of a recent study by Di Martino et al. [23], which showed a trend, although not statistically significant, toward improved diagnostic accuracy with gadoxetate disodium-enhanced MR imaging compared with multidetector CT for the detection of HCC particularly for smaller lesions, particularly those less than 2 cm. In particular, our data showed that 4 HCCs (mean size, 10 mm) were detected, correctly, only with gadoxetate disodium-enhanced MR imaging.

Our results are in opposition with findings of [24, 25]. In fact, Watanabe et al. [24] report in the conclusion that the incorporation of hepatocyte phase images did not improve the diagnostic accuracy of gadoxetate disodium-enhanced MRI for locally recurrent HCCs after RFA because the reactive therapeutic response induced pseudolesions with similar findings; the possible presence of these pseudolesions should be recognized by radiologists as a pitfall when interpreting gadoxetate disodium-enhanced MR images after RFA, especially by inexperienced radiologists. Motosugi et al. [25] mentioned that over 10% of vascular pseudolesions, such as arteriovenous shunts, show hypointensity in the hepatocyte phase and can be misinterpreted as HCC even with the combined interpretation of hepatobiliary and dynamic contrast enhanced MRI. 

These findings can be explained because in this study the interpretation of radiological data (residual tumor or necrosis) is the result of a combined assessment of the different vascular phases with the addition of the hepatospecific phase; therefore, an arterio-venous shunt or a vascular pseudolesion presents a similar signal to vessels, easily recognizable by an expert radiologist. The arteriovenous shunt or a vascular pseudolesion show a different signal from the residual tumor and tissue necrosis: in our series the residual of HCC shows a washin and wash-out with constant hypointense signal in the hepatospecific phase; an area of necrosis shows constant hypointense signal in the different phases of the study including hepato-specific phase; arteriovenous shunt or a vascular pseudolesion shows isointense signal compared to the vessels.

Our study has a number of limitations. The reviewers had been involved in the original studies, and this may have determined a recall bias. However, given the long time interval between the exams and the revision sessions, we believe that this recall bias was minimized. No correlation was obtained with diffusion-weighted MR data. This was done to compare the dynamic phases of CT and MRI. Diffusion MRI offers relevant additional information, and now we have started correlating the diffusion imaging with the dynamic imaging of MRI. 

In summary, the results of our study indicate that combined interpretation of dynamic and hepatobiliary phase MR images improves the diagnostic accuracy of gadoxetate disodium-enhanced MR imaging for the detection of residual HCC and new HCC in treated patients compared with either dynamic MR or CT alone.

## Figures and Tables

**Table 1:** Demographics, etiology of cirrhosis, and clinical stage of the 34 patients.

Patients	Score
Age (mean ± standard deviation)	60 ± 10
Sex (male/female)	2/1
Aetiology	
Virus B	14
Virus C	10
Alcohol	6
Others	4
Child-pugh	
A	8
B	25
C	1
Alpha-fetoprotein (ng/mL)	40–100

**Table 2:** One-month followup results.

	Sensitivity	Specificity	Positive predictive value	Negative predictive value	*P* value
CT	6/12 (50%)	30/32 (94%)	6/8 (75%)	30/36 (83%)	<0.01
MRI	11/12 (92%)	31/32 (97%)	11/12 (92%)	31/32 (97%)	<0.001

**Table 3:** Three-month followup results.

	Sensitivity	Specificity	Positive predictive value	Negative predictive value	*P* value
CT	9/16 (56%)	30/32 (94%)	9/11 (82%)	30/37 (81%)	<0.01
MRI	16/16 (100%)	31/32 (97%)	16/17 (94%)	31/31 (100%)	<0.001

**Table 4:** CT score at first (1 M) and three months (3 M) in arterial phase (AP) and portal phase (PP) for false positive.

False positive	AP CT score	PP CT score
1 M	4	4
	4	4

3 M	4	3
	4	3

**Table 5:** MR score at first (1 M) and three months (3 M) in arterial phase (AP), portal phase (PP), and hepatobiliary phase (HP) for false positive.

False positive	AP MR score	PP MR score	HP MR score
1 M	4	4	3
3 M	4	3	2

**Table 6:** CT and MR score at first months (1 M) at arterial phase (AP), portal phase (PP), and hepatobiliary phase (HP) for new HCCs.

New HCC 1 M	AP CT score	PP CT score	AP MR score	PP MR score	HP MR score
1	1	1	3	3	4
2	1	1	2	3	4

**Table 7:** CT and MR score at three months (3 M) at arterial phase (AP), portal phase (PP), and hepatobiliary phase (HP) for new HCCs.

New HCC 3 M	AP CT score	PP CT score	AP MR score	PP MR score	HP MR score
1	4	4	4	4	4
2	4	4	4	4	4
3	1	1	2	3	4
4	1	1	2	3	4
5	1	1	4	4	4
6	1	2	3	3	4
